# Mechanical Characterization of MWCNT-Reinforced Cement Paste: Experimental and Multiscale Computational Investigation

**DOI:** 10.3390/ma16155379

**Published:** 2023-07-31

**Authors:** Ioannis E. Kavvadias, Konstantinos Tsongas, Kosmas E. Bantilas, Maria G. Falara, Athanasia K. Thomoglou, Fani I. Gkountakou, Anaxagoras Elenas

**Affiliations:** 1 Department of Civil Engineering, Democritus University of Thrace, 67100 Xanthi, Greece; kbantila@civil.duth.gr (K.E.B.); mfalara@civil.duth.gr (M.G.F.); athomogl@civil.duth.gr (A.K.T.); fgkounta@civil.duth.gr (F.I.G.); elenas@civil.duth.gr (A.E.); 2 Department of Industrial Engineering and Management, International Hellenic University, 57001 Thessaloniki, Greece; k.tsongas@ihu.edu.gr

**Keywords:** carbon nanotubes, MWCNTs, cement paste, finite element modeling, multiscale modeling, representative volume element, random sequential adsorption algorithm

## Abstract

Computational approaches could provide a viable and cost-effective alternative to expensive experiments for accurately evaluating the nonlinear constitutive behavior of cementitious nanocomposite materials. In the present study, the mechanical properties of cement paste reinforced with multi-wall carbon nanotubes (MWCNTs) are examined experimentally and numerically. A multiscale computational approach is adopted in order to verify the experimental results. For this scope, a random sequential adsorption algorithm was developed to generate non-overlapping matrix-inclusion three-dimensional (3D) representative volume elements (RVEs), considering the inclusions as straight elements. Nonlinear finite element analyses (FEA) were performed, and the homogenized elastic and inelastic mechanical properties were computed. The use of a multiscale computational approach to accurately evaluate the nonlinear constitutive behavior of cementitious materials has rarely been explored before. For this purpose, the RVEs were analyzed both in pure tension and compression. Young’s modulus as well compressive and tensile strength results were compared and eventually matched the experimental values. Moreover, the effect of MWCNTs on the nonlinear stress–strain behavior of reinforced cement paste was noted. Subsequently, three-point bending tests were conducted, and the stress–strain behavior was verified with FEA in the macro scale. The numerical modeling reveals a positive correlation between the concentration of MWCNTs and improved mechanical properties, assuming ideal dispersion. However, it also highlights the impact of practical limitations, such as imperfect dispersion and potential defects, which can deteriorate the mechanical properties that are observed in the experimental results. Among the different cases studied, that with a 0.1 wt% MWCNTs/CP composite demonstrated the closest agreement between the numerical model and the experimental measurements. The numerical model achieved the best accuracy in estimating the Young’s modulus (underestimation of 13%), compressive strength (overestimation of 1%), and tensile strength (underestimation of 6%) compared to other cases. Overall, these numerical findings contribute significantly to understanding the mechanical behavior of the nanocomposite material and offer valuable guidance for optimizing cement-based composites for engineering applications.

## 1. Introduction

Cementitious composites reinforced at the nano scale have gained much interest in building construction in the last decades. Their great potential in mechanical properties as well as their high performance in durability have attracted the interest of researchers for further in-depth investigation. However, cement-based materials are considered inherently brittle, with inefficient tensile strength and low toughness, leading to the deterioration of fracture resistance. Multi-wall carbon nanotubes (MWCNTs) can be incorporated into the cement to enhance the mechanical properties, preventing crack formation into the cementitious matrix.

The addition of nano-scale fibers into cement paste has turned out to be an effective way to reinforce cementitious materials. More specifically, it has been reported that adding an extremely low amount of MWCNTs ranging between 0.01 and 0.02 wt% of the binder, in contrast to higher amounts (greater than 0.03 wt%), can provide strength enhancement to the materials [1]. Regarding the CNTs’ aspect ratio, some progresses has been achieved, including the effects of different shapes of carbon fibers on the piezoresistivity, and it was concluded that conductive filler with a high aspect ratio of about 600 could provide better mechanical performance and piezoresistive sensitivity [2]. Cerro-Prada et al. [3] investigated the use of MWCNTs characterized by a high aspect ratio of about 1050 and concluded that the incorporation of 0.01 wt% MWCNTs in cement paste yielded 1.6% and 4.9% greater compression and flexure performance, respectively, as well as 11% resistivity degradation compared to the reference sample. Konsta-Gdoutos et al. [4] investigated the effect of incorporating 0.1 wt% short MWCNTs of different types and aspect ratios ranging from 200 to 330 on mechanical performance (compressive strength, tensile strength, and elastic modulus). Adding MWCNTs had a minimal effect on compressive strength, i.e., only 5%. However, the effect of MWCNTs on the elastic modulus and flexural strength was significant, as these were enhanced by 87–120% for all types of MWCNTs.

Other researchers have studied the effect of different aspect ratios between long (1250–3750) and short (157) MWCNTs, concluding that a cementitious matrix incorporating a low concentration (0.1 wt%) of long MWCNTs exhibits a similar mechanical performance as those incorporating a higher concentration (0.2 wt%) of short MWCNTs [5]. Similar results were presented by Konsta-Gdoutos et al. [6], who concluded that the percentage of 0.048 wt% high-aspect ratio (1600) MWCNTs and 0.08 wt% low-aspect ratio (700) MWCNTs increased the flexural strength of reinforced cement paste by 25% in contrast to plain cement paste. Moreover, Metaxa et al. [7] incorporated 0.2 wt% short MWCNTs (aspect ratio = 153) into the mortar, which resulted in a 60% electrical resistivity decrease; however, a 25% and 10% increase was observed in flexural and compressive strengths, respectively.

Despite extended experimental research on cement-based composites, especially reinforced with CNTs, there are limited numerical approaches dealing with this issue, particularly adopting 3D computational modeling [8,9,10,11,12]. Analytical and semi-analytical homogenization models have commonly been used to evaluate the effective mechanical properties of composites in the past years [13,14]. However, these models cannot handle the geometry variability governing a composite material. Thus, computational homogenization methods combined with multiscale FEM simulations have gained increasing attention recently [15,16,17]. Another advantage of this method is the adequate description of the micromechanical stress–strain state. FEM modeling based on a representative volume element (RVE) provides a wide versatility regarding the microstructure configuration. Thus, this modeling approach has been implemented to investigate the effective material properties of composite materials. Recently, multiscale analyses have been also conducted incorporating stochastic procedures [18], as well as machine-learning algorithms [19]. The vast majority of multiscale investigations on composite materials examine their elastic properties, concluding with bulk-effective properties. On the other hand, few studies adopt nonlinear homogenization techniques to deal with the nonlinear behavior of composite structures [12,20].

In the present study, the mechanical properties of cement paste reinforced with MWCNTs are examined both experimentally and numerically. A multiscale computational simulation was adopted in order to verify the experimental results. For this scope, 3D RVEs were constructed using a random sequential adsorption algorithm, and finite element analyses were performed. Apart from calculating the homogenized effective elastic properties, the nonlinear constitutive behavior of the material in both tension and compression was accurately assessed. For this scope, a sophisticated material model was adopted. Finally, macro-scale modeling was performed, reproducing a three-point bending experiment on a real scale. The homogenized elastic and inelastic properties of CP/MWCNTs composites aligned well with experimental results. MWCNTs enhanced strength in tension and compression, with improved deformation capacity. However, computational analysis cannot determine the deterioration of these properties in the case of higher MWCNT concentrations. Nonetheless, the computational approach accurately assessed effective elastic properties and nonlinear behavior in tension and compression in low concentrations. Specifically, for 0.1 wt% MWCNTs/CP, the numerical model underestimated Young’s modulus by 13%, overestimated compressive strength by 1%, and underestimated tensile strength by 6%. The numerical insights provided here offer valuable guidance for optimizing cement-based composites, aiding in understanding the mechanical behavior of the nanocomposite material. These cost-effective evaluations are beneficial for various engineering applications in which the structural integrity should be ensured.

## 2. Materials and Methods

### 2.1. Experimental Work

#### 2.1.1. Materials

Cementitious pastes were produced using Type I ordinary Portland cement (OPC) 42.5 R provided by TITAN HELLAS (Table 1), and the pastes were modified with MWCNTs. The MWCNTs, provided by Nanocyl in powder form, have a diameter of 9.5 nm, length of 1.5 μm, and density of 1.66 gr/cm^3^. The CNTs’ dispersion is the key factor in the manufacturing process to optimize their performance and improve their incorporation into the cement paste [21]. In the present research, sodium dodecylbenzene sulfonate (SDBS) by Aldrich and Tri-n-butyl phosphate (TBP) were added to the mixture to achieve better dispersion of the MWCNTS [22].

**Figure 1:**
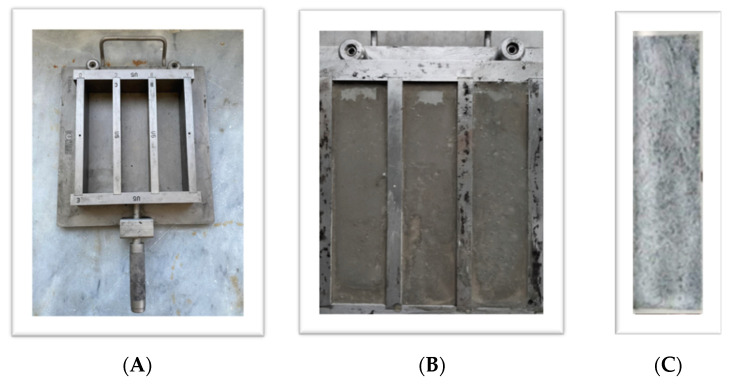
(**A**) Steel molds for matrix casting after a mixture; (**B**) cement matrix poured in the molds; and (**C**) cured specimen after demolding.

Ultrasonication energy was applied to break up MWCNT agglomerates into the aqueous solutions. A 500 W high-intensity ultrasonic apparatus equipped with a 19 mm diameter probe was used to impose ultrasonic energy for 60 min [23,24]. A temperature controller up to 60 °C was also used to avoid the solutions’ overheating. Upon completing the above procedure, the MWCNTs, SDBS, and TBP aqueous solutions were inserted into the Portland cement, with a proportion of water-to-cement ratio w/c = 0.5. In total, specimens with 4 amounts of MWCNTs per weight of cement were constructed. Finally, the casting of the specimens was performed using steel molds (Figure 1).

#### 2.1.2. Mechanical Testing Procedure

**Figure 2:**
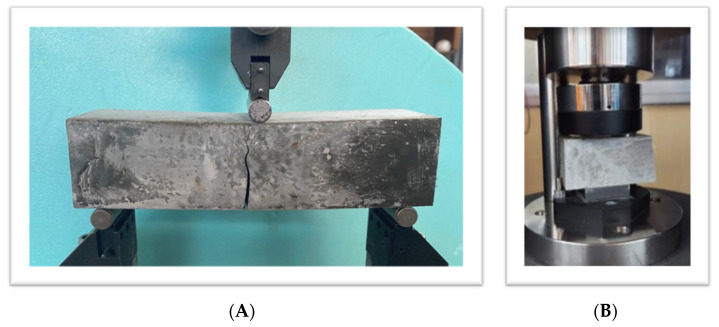
Experimental test setup of (**A**) three-point bending and (**B**) compressive strength of the nano-reinforced cement paste specimens.

A series of three-point bending and compression experiments were performed to evaluate the flexural and compressive strength of the MWCNT-modified cement pastes. The dimensions of the test specimens were 40 mm × 40 mm × 160 mm. Flexure tests were performed, according to the ASTM C348 [25], in an MTS servo-hydraulic closed-loop machine that is dynamically rated to handle loads varying from 1 to 25 kN under displacement control; the displacement rate was kept at 0.1 mm/min. To complement the setup, strain gauges with a parallel aid of 1 mm were utilized, providing valuable data on the specimen’s deformation during the test All nanocomposite specimens were examined at room temperature with three repetitions for each concentration, in order to extract reliable results with the minimum standard deviation. After the flexural test procedure, the halves of every specimen, 6 per each concentration of MWCNTs/CP nanocomposite specimens, were subjected to compression, following the ASTM C349 [26] guidelines. The compressive strength was determined by utilizing a 3000 kN load cell. The tests were performed under force control, and the loading rate was kept at 0.3 kN/s. It should be noted that all the tests were performed with specimens at the age of 28 days. Figure 2 depicts the experimental setups.

### 2.2. Numerical Multiscale Investigation

#### 2.2.1. RVE Generation

**Figure 3:**
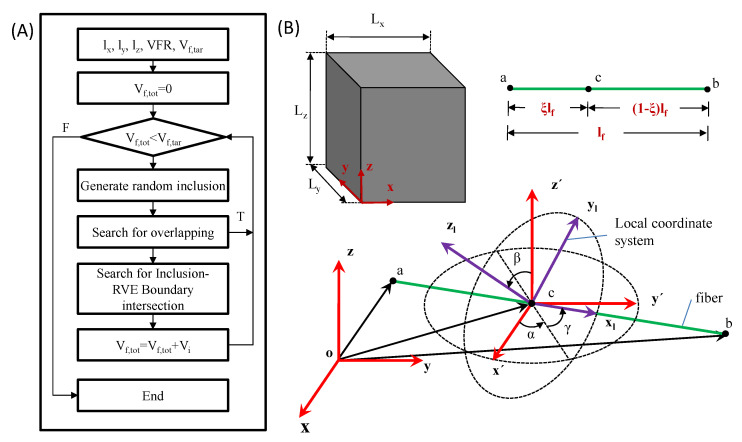
(**A**) Flowchart of the random sequential adsorption algorithm and (**B**) fiber spatial distribution.

To investigate the mechanical properties of the MWCNT-modified cement pastes in multiple scales adopting computational homogenization, the generation of the representative volume element (RVE) that corresponds to the problem is of utmost significance. Therefore, for the scope of this study, an algorithm that produces the geometry of the RVEs was developed. In order to generate non-overlapping matrix-inclusion RVEs, the random sequential adsorption method was adopted [27]. Specifically, given the dimensions of the cuboid-shaped RVE (l_x_, l_y_, and l_z_), the dimensions of the nanotube (length and diameter), and the volume fraction (vol%) of the nanotubes, the total volume of the nanotubes within the RVE is calculated. Subsequently, using a repetitive procedure, the nanotubes are placed successively with random orientation into the RVE until the total volume of the fibers (V_f,tot_) becomes equal to the required volume (V_f,tar_). As it is crucial to avoid overlapping, each newly added nanotube undergoes thorough scrutiny to ensure it does not intersect with any previously placed nanotubes. In the case in which an overlap is detected, the inclusion is discarded, and the process is repeated by generating and placing a new randomly oriented inclusion. If no instances of overlap are identified, the inclusion is considered valid. Subsequently, an examination of potential intersections between the inclusion and the surfaces of the RVE is conducted. If an intersection with a face is detected, the parts of the inclusion outside of the RVE are removed, and the volume of the inclusion (V_i_) is recalculated, taking into account the part inside the RVE. The algorithm can be summarized in Figure 3A.

To construct randomly distributed inclusions, the start and the end points of the centroid line of every inclusion are determined using a random number generator. Initially, the coordinates of a random point (x_c_, y_c_, z_c_) are calculated throughout the length of each nanotube. These coordinates follow the uniform distribution inside the RVE boundaries. Subsequently, also following the uniform distribution, the random variable ξ, ranging from 0 < ξ < 1, is determined. The value of ξ designates the location of point c throughout the fiber. Finally, the fiber is parallel to the x_l_ axis at the local coordinate system x_l_y_l_z_l_, with the axis origin the point c. The local coordinate system x_l_y_l_z_l_ results from successive rotations α, β, and γ of an x’y’z’ coordinate system, and the x’y’z’ coordinate system is defined by shifting the global coordinate system xyz by the vector **oc** (Figure 3B). The angles α, β, and γ are called Euler angles. The angles α ranges from −π to π, and angle β ranges from −π/2 tο π/2. The values of all angles follow a uniform distribution. As such, points a (p_a_) and b (p_b_) are given by the following relations (Equation (1a) and (1b)):

(1a) $$p_{a}={\left(R_{3}R_{2}R_{1}\right)}^{T}\left[\begin{array}{c} -\xi l_{f}\\ 0\\ 0 \end{array}\right]\;+\;\left[\begin{array}{c} x_{c}\\ y_{c}\\ z_{c} \end{array}\right]\;$$

(1b) $$p_{b}=\;{\left(R_{3}R_{2}R_{1}\right)}^{T}\left[\begin{array}{c} (1-\xi )l_{f}\\ 0\\ 0 \end{array}\right]\;+\;\left[\begin{array}{c} x_{c}\\ y_{c}\\ z_{c} \end{array}\right]$$

where l_f_ is the length of the fiber, and **R**_1_, **R**_2_, and **R**_3_ are the rotation matrix corresponding to the Euler angles (Equation (2)):

(2) $$R_{1}=\left[\begin{array}{ccc} \cos\alpha & \sin\alpha & 0\\ -\sin\alpha & \cos\alpha & 0\\ 0 & 0 & 1 \end{array}\right],\;\;\;R_{2}=\left[\begin{array}{ccc} 1 & 0 & 0\\ 0 & \cos\beta & \sin\beta \\ 0 & -\sin\beta & \cos\beta \end{array}\right]\;,\;\;\;and\;\;R_{3}=\left[\begin{array}{ccc} \cos\gamma & \sin\gamma & 0\\ -\sin\gamma & \cos\gamma & 0\\ 0 & 0 & 1 \end{array}\right]\;\;$$

**Figure 4:**
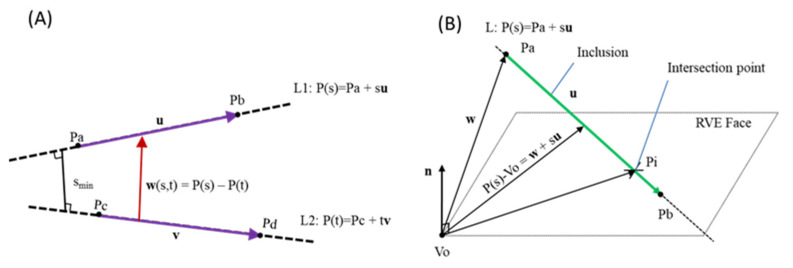
Intersection distance control between (**A**) inclusions and (**B**) inclusion and surface.

In order to check possible overlaps among the fibers, the closest point of approach (CPA) method is adopted (Figure 4A) [28]. The fibers are considered 1D elements during this method due to their high aspect ratio. The minimum distance between two fibers (w_min_) is calculated and compared with the minimum accepted predefined value S_tol_. In the case of w_min_ < S_tol_, the new inclusion is rejected. In the current study, the S_tol_ is assumed to be equal to the diameter of the examined fibers. If no overlap is detected between the inclusion and any of the previously placed nanotubes, the possible intersection of the inclusion with the RVE faces is investigated (Figure 4B). The intersection point between the centroid line (inclusion) and the RVE face is calculated by solving Equation (3):

(3) $$n\cdot \left(w+s_{i}u\right)=0$$

where **n** is the vector of the face plane. In case the s_i_ parameters take values s_i_ < 0 or s_i_ > 1, the inclusion does not intersect the plane. On the other hand, the inner product c = (**P_a_** − **P_i_**)∙**n** is calculated. When c >0, the point P_b_ is outside the RVE; therefore, the section P_i_-P_b_ is removed. Otherwise, if c < 0, the section P_i_-P_a_ is removed. This procedure is repeated for any face of the RVE. Since the inclusion sections outside the RVE are removed, the inclusion’s volume (V_i_) is added to the placed fibers’ total volume (V_f,tot_).

The algorithm described above was generated in MATLAB [29]. Given the dimensions of the RVE (l_x_, l_y_, and l_z_), the length (l_f_), and the aspect ratio (a_R_) and volume fraction (vol%) of the nanotubes to the matrix, the algorithm generates the geometry of the RVE in .dwg format. Subsequently, the geometry is transformed into “.iges” format in order to be imported to the FEM software and proceed to the analyses.

#### 2.2.2. Finite Element Modeling

In order to validate the linear and nonlinear properties of the composite material as the experimental procedure calculated them, the generated RVEs were properly simulated and analyzed by exploiting computational homogenization. ANSYS™ software (ANSYS, Inc., Canonsburg, PA, USA) [30] was used to investigate the mechanical behavior in a two-level process: nano and macro. The explicit dynamic module was employed to simulate the response of specimens accurately. This process captures their linear and nonlinear material behavior considering different fracture modes. For this scope, the Riedel–Hiermaier–Thoma (RHT) [31,32] plasticity model, initially introduced for simulating concrete materials, was adopted to simulate the cement paste. The RHT constitutive model is a plasticity damage model incorporating a shear failure, able to represent in detail the nonlinear behavior of cement pastes reinforced with MWCNTs. Furthermore, as far as the authors are aware, though this material is frequently employed to simulate concrete members, its application in multiscale approaches for cement paste nanocomposites has not been observed. The experimental results of the plain cement paste were used to calibrate the model of the matrix material of the RVEs. Although the cement paste was simulated using solid finite elements, the nanotubes were simulated using 1D elements [33] with circular cross-section and geometry characteristics identical to the MWCNTs used in the experimental work. Moreover, the MWCNTs were simulated as a linear elastic material until failure.

It is essential to introduce the proper periodic boundary conditions in the generated RVE to homogenize the effective properties of the examined composites reinforced with dispersed nanotubes. Due to the fact that nanotubes are simulated as 1D elements, the mesh between the matrix and the inclusions was independent. As such, the matrix of the RVE can be meshed periodically [34]. Specifically, the mesh algorithm ensures that the nodes of each vertex or surface align with the nodes of the corresponding geometries on the opposite side [35]. To this end, the corners and specific nodes at the vertexes and the faces of the RVE are properly constrained [36].

Subsequently, the three-point bending experiment was reproduced by FE analysis on a real scale. The specimens in the macro scale were modeled with one bulk material. Specifically, the RHT material was used with properties dependent on the concentration of MWCNTs. Since the homogenized properties are calculated by the RVEs analyses, the effective Young’s modulus, the compression, and the tension strength were defined. Moreover, given the force–displacement behavior of the RVEs under uniaxial tension and compression, the material parameters that affect the nonlinear behavior of the bulk material used in the macro scale were selected.

## 3. Results

### 3.1. Nanoscale: RVE Analysis

**Figure 5:**
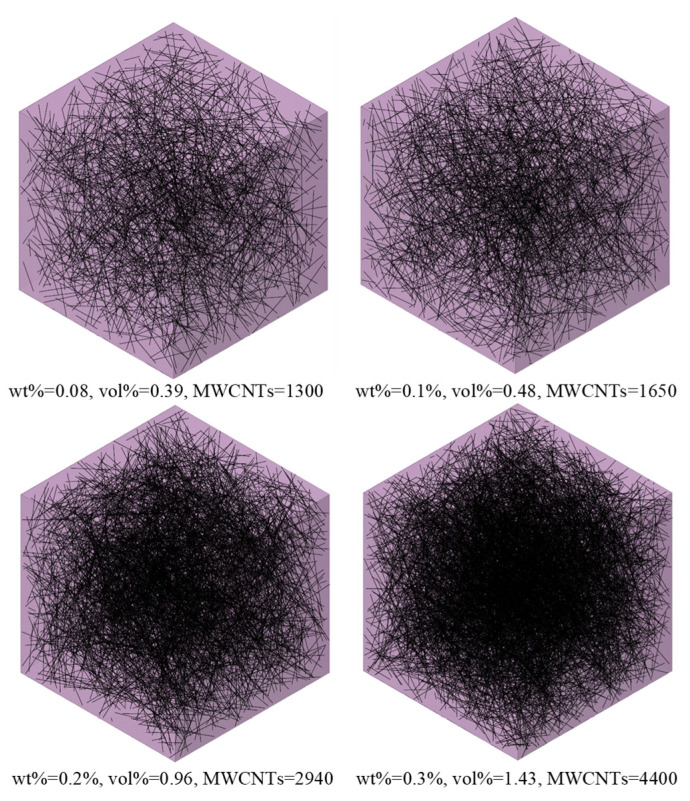
Indicative RVE geometries for the examined content of MWCNTs in cement paste.

Samples with a weight fraction of nanotubes wt% = 0.08, 0.1, 0.2, and 0.3 were constructed and studied. The MWCNTs have a length l_f_ = 1.5μm and a diameter d = 9.5 nm, resulting in an aspect ratio a_R_ = 158. The constructed RVEs were selected to be of cubic shape with size l_x_ = l_y_ = l_z_ = 2L_f_ = 3 μm, based on the properties of the nanotubes. Based on the literature, RVEs with side length l ≥ 2l_f_ result in reliable results [37]. As such, due to increasing computational demands, as the size of the RVE increases [38], l = 2l_f_ was selected. Due to the fact that the RVE generation algorithm is based on the volume fraction of the nanotubes, the weight fraction (wt%) is converted to the volume fraction (vol%). Indicative RVEs for the considered rates are presented in Figure 5.

For the appropriate simulation, the mechanical properties of the two basic materials, the nanotubes and the cement paste, were defined with the material laws stated in the previous section. The MWCNTs’ Young’s modulus is equal to E = 1000 GPa, with a stress limit of up to 50 GPa failure [39]. Regarding the matrix material, Young’s modulus Ε = 11,300 MPa, a flexural strength σ_f_ = 5.9 MPa, and a compressive strength σ_c_ = 52.4 MPa were set as calculated from the three-point bending and the uniaxial compression test of the plain cement paste. Considering that no imperfections were introduced in the simulation, the flexural strength under uniaxial bending, which results from the failure of the extreme bottom fibers of the specimen’s cross-section due to tension, can be considered equal to tensile strength (σ_t_). In this way, it is sufficient to study the RVEs in pure compression and pure tension in order to calculate their elastic and inelastic mechanical properties. Based on this, the rest parameters of the RHT model are properly selected after an iterative process concluding with an acceptable agreement between the calibrated model and the experimental results in terms of stress–strain behavior of the material during three-point bending and uniaxial compression. The top plate of the RVE was subjected to a controlled displacement in the longitudinal direction, and periodic boundary conditions were set to all nodes with relevance to each other on the opposite surfaces. At the same time, it is important to note that the displacements of the bottom plate were constrained only in the vertical direction. The specific values chosen for this imposed displacement were carefully selected to replicate the ultimate strain observed in experimental tests.

Mesh sensitivity analyses were conducted using convergence studies on a normalized elastic modulus, as suggested by the literature, to obtain mesh-independent results [40]. The studies revealed that convergence was achieved with nearly 23,000 elements for the RVE with 0.08 wt% WMCNTS, whereas 72,000 elements are required in the case of 0.3 wt% MWCNTs. Hexahedral elements “SOLID 186” were utilized for the matrix’s solid part, and “REINF264- type elements were used to simulate the line bodies that correspond to the MWCNTs.

**Figure 6:**
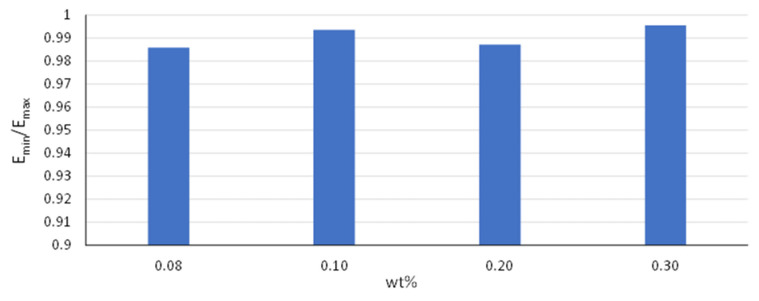
Young’s modulus ratio (E_min_/E_max_) under uniaxial loading for the different RVEs.

The composite material, consisting of randomly oriented nanotubes, can be assumed to present an isotropic behavior [41]. To verify this characteristic, 100 RVEs are constructed for each weight fraction of nanotubes. Every RVE is examined under uniaxial compression in every direction. In any case, the elastic modulus E parallel to the loading is calculated. Subsequently, the ratio of E_min_/E_max_ is calculated for each model. As presented in Figure 6, this ratio took values over 0.98 in every case. Thus, the behavior of the examined composite materials can be assumed as isotropic for any of the considered weight fractions, given the uniform distribution of the randomly oriented nanotubes in the matrix.

**Figure 7:**
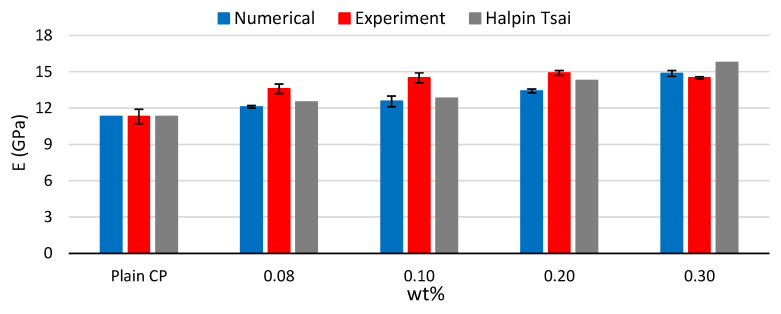
Comparison among numerical, experimental, and micromechanical model calculation of Young’s modulus for the plain CP and the different CP/MWCNT nanocomposite specimens.

The experimental values are obtained following the ASTM C348 guidelines. To this end, three specimens need to be examined for each case. From the results, the mean values and their standard deviation are calculated. As such, even in the case of the numerical investigation, the results of three different RVEs are reported and compared to the experimental ones for each concentration of MWCNTs. In Figure 7, Young’s modulus’s mean values and standard deviation are presented comparatively. Additionally, the results of the Halpin–Tsai model [42], which is suitable for randomly distributed fibers, are demonstrated.

It can be observed that the results of both the numerical analyses as well as the micromechanical model underestimate Young’s modulus in every case, except for wt% = 0.30. In the case of wt% = 0.30, the experimental measurements result in a mean value even lower than the case of wt% = 0.20. This could be related to inadequate dispersion of the MWCNTs on the matrix material [43]. Obviously, in the numerical analyses, imperfections like this cannot be attributed; thus, Young’s modulus increases as the content of the MWCNTs increases. This is also the case regarding the micromechanical model. Additionally, it should be noted that the results obtained by the numerical analyses are close enough to those calculated via the Halpin–Tsai model. The maximum increase of 32% was measured experimentally in the case of wt% = 0.20, and based on the numerical analysis and the micromechanical model, increases of 19% and 27% were calculated, respectively. Comparable increasing ratios have been reported by Al-Rube et al. [5]. Interestingly, the standard deviation between the experimental and the numerical results are of the same scale, without exhibiting a specific trend. In both instances, the maximum standard deviation amounts to 0.4 GPa. In the case of the plain cement paste, the standard deviation is not reported by the numerical analyses, as the result obtained by a specific model, consisting only of the matrix model, could also serve as a consistency test for the material modeling of the matrix. The material modeling was calibrated using the mean values of the plain CP; thus, the measurements coincide.

For the determination of the inelastic properties, the RVEs are analyzed both in pure tension and compression due to the characteristics of the inelastic behavior of the cementitious matrix. Explicit dynamic analyses are performed to exploit the RHT material model to capture the nonlinear behavior of the CP/MWCNTs. The properties of the plain cement paste, the matrix material, are appropriately calibrated based on the experimental results. Due to the reduced experimentally measured Young’s modulus of 0.30 wt% MWCNTs, the nonlinear behavior will be evaluated for the cases of wt% = 0.08, 0.1, and 0.2 MWCNTs. The experimentally measured reduced Young’s modulus implies imperfections in the specimen that affect the strength significantly—a phenomenon that cannot be captured in the analysis. Thus, the nonlinear mechanical behavior of the CP with a MWCNT concentration of wt% = 0.30 is not examined computationally.

**Figure 8:**
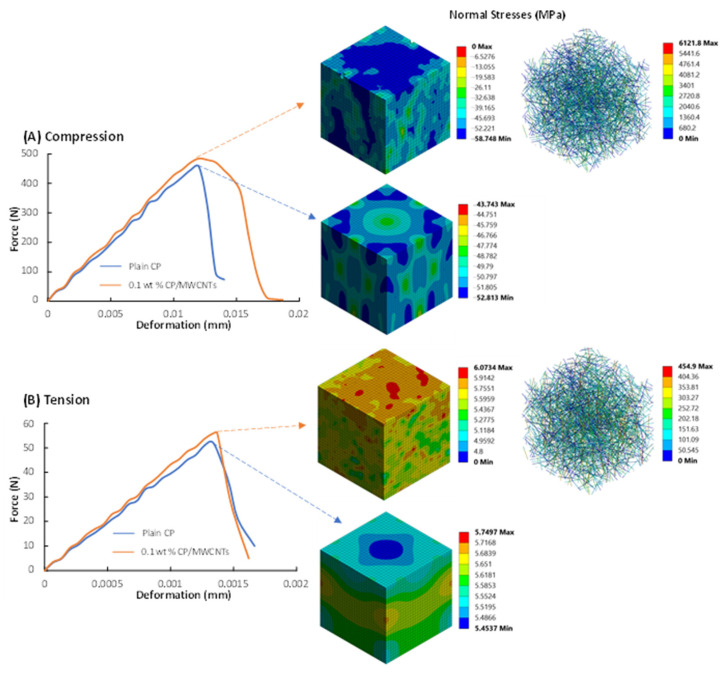
Force–displacement curves and normal stress distribution in MPa, corresponding to the maximum force for the plain CP and the CP/MWCNT nanocomposite specimens under uniaxial compressive and tensile loading.

Figure 8 presents force–displacement curves for the plain CP and CP/MWCNTs with concentration wt% = 0.1. The curves are calculated computationally under uniaxial compression and tension. In addition, the stress distributions corresponding to the maximum load capacity are illustrated for every RVE. It can be observed that the numerical model, apart from the increase in Young’s modulus, also captures the increased strength both in tension and compression. Interestingly, it is observed that the increase in the strength, along with the increase in the elastic modulus, also results in higher ultimate deformation. Moreover, under compression, the MWCNTs also affect the nonlinear behavior by increasing the nonlinear deformation capacity. Since only the ultimate compressive strength was captured during the experimental compression tests of this work, the whole force–deformation response is compared to other works. Similar compressive behavior has been reported elsewhere [44], which verifies the efficiency of the FE model to calculate the stress–strain behavior of CP/MWCNT specimens.

**Figure 9:**
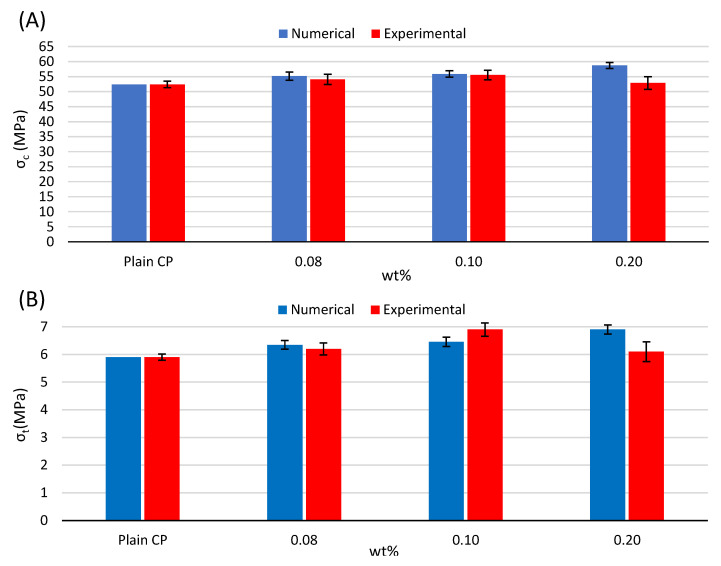
Comparison between numerical and experimental (**A**) compressive and (**B**) tensile strength for the plain CP and the different content of MWCNTs/CP nanocomposite specimens.

The tensile and compressive strengths for all the MWNCT concentrations are summarized in Figure 9. Based on the experimental results, the strength increases in the case of 0.08 and 0.1 wt% MWCNTs/CP. However, in the case of 0.2 wt%, the compressive strength is lower than that with 0.1 wt% MWCNT concentration, and the tensile tends also to decrease compared with the lower concertation of MWCNTs. This observation is a result of crack formation in stress concentration areas due to the presence of agglomerates and bundles of MWCNTs around cement particles obstructing the hydration process and resulting in a weak structure. This feature cannot be attributed to the adopted numerical modeling. However, in the case of lower MWCNT concentrations, the strengths are well estimated by the numerical simulation. Specifically, the compressive strength is slightly overestimated, and the tensile strength is underestimated only in the case of 0.1 wt% WMCNTs. For the cases with 0.08 wt% and 0.1 wt% MWCNTs/CP, the compressive strength exhibited a 3% and 6% increase, respectively, compared to the plain cement paste. Meanwhile, the numerically calculated strengths were even higher, showing a 5% and 7% enhancement over the strength of the plain cement paste. On the other hand, for the same concentrations, the experimental measurements showed an increase in tensile strength of 5% and 17%, respectively. However, the numerical analyses yielded slightly different results, calculating an 8% increase in tensile strength for the case of 0.08 wt% MWCNTs/CP and a 9% increase in tensile strength for the case of 0.1 wt% MWCNTs/CP. These values are in good agreement with those reported by Cerro-Prada et al. [3]. In any case, the variability in the results, expressed in terms of standard deviation and illustrated with error bars, is slightly lower in the case of numerical analyses, with values of 1.6 GPa and 0.24 GPa for the compression and tensile strengths, respectively, compared to the experimental values of 1.1 GPa and 0.17 GPa for the compression and tensile strengths, respectively. Due to the fact that the matrix material is calibrated based on the mean experimental results of the plain CP, the numerical analysis results are obviously expected to be identical to the experimental ones.

### 3.2. Macroscale: Three-Point Bending Tests and Analysis

**Figure 10:**
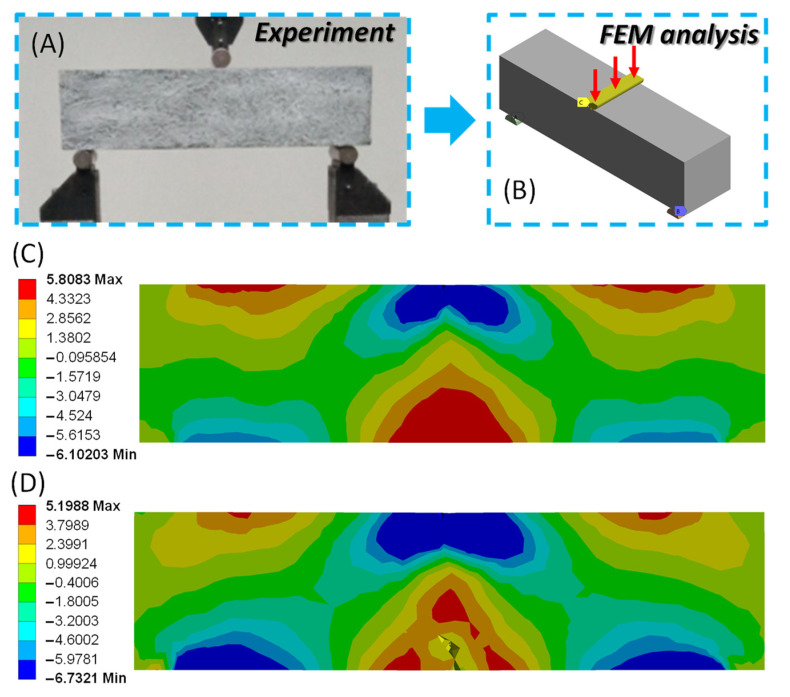
(**A**) Experimental setup for flexural tests; (**Β**) simulation model of the specimen under bending; (**C**) normal stress distributions in MPa before failure due to tensile stresses; (**D**) normal stress distributions in MPa after crack opening.

A macro-scale-level simulation examined the bending behavior of the CP/MWCNT nanocomposite specimens. The three-point bending tests were carried out as shown in Figure 10A. The FEM simulation accurately captures the experimental setup’s geometry and boundary conditions (Figure 10B). The experimental setup consists of two supports positioned 1 cm inward from the edges of the 40 mm × 40 mm × 160 mm specimen, along with a centrally located loading pin. The specimens were modeled using the RHT material model with effective material properties presented in the previous section, and the supports and the loading pin were simulated with a linear elastic material with Young’s modulus E = 200 GPa. Subsequently, a frictionless model was adopted to simulate the contact areas between the specimen, the supports, and the loading pin. Finally, a vertical velocity was applied in increments to the top pin to reproduce the experimental testing. The mesh sensitivity analyses revealed that convergence was achieved with nearly 100,000 elements, of the “SOLID 187” type, for the bending specimens.

Figure 10C,D show the stress distributions on the face of the specimen before and just after failure. The failure occurred as a result of surpassing the tensile strength on the lower surface of the cross-section in the middle of the beam’s span. It can be observed that the model can reproduce a realistic crack pattern. This fact implies that the numerical simulation captures the behavior of CP/MWCNT composites under three-point bending testing both qualitatively and quantitatively.

**Figure 11:**
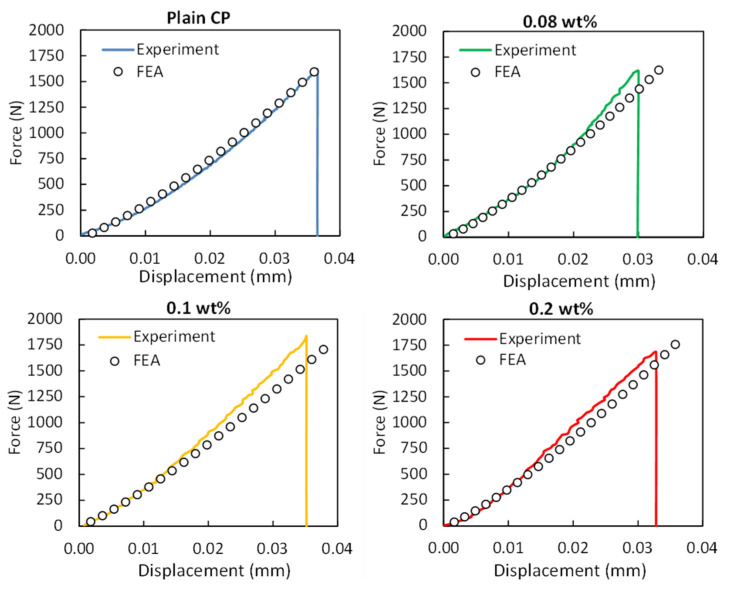
Comparison between FEA generated and experimental force–displacement curves of the CP/MWCNT nanocomposite specimens under bending.

The resulting reaction force at the bottom was obtained, considering a fixed boundary condition on the bearing pins. As such, the force–displacement results measured during the experiments were compared with the numerical results. It has to be mentioned that in the numerical models, the materials were simulated with the mean values based on the analyses of the RVEs, whereas the compared experimental results are those with the lower deviation from the experimental mean values. The force–displacement behavior of the CP/MWCNT nanocomposite specimens is demonstrated in Figure 11, showcasing a notable association between the experimental flexural tests and the force–displacement data derived from the FEA simulations. Nevertheless, it is crucial to acknowledge that as displacement increases, the experimental curves deviate further from the FEA simulation due to the considerable influence of cement paste defects on the bending response. The differences in the elastic modulus and tensile strength, as captured by the RVEs analyses, are also evident in the case of the macro-scale results. The elastic modulus is slightly underestimated (Figure 7), and in the case of the flexural strengths, the results follow the strength under pure tensile (Figure 9). A complete agreement of the results is observed only in the case of plain cement paste specimens. The material model parameters were analyzed to minimize the difference between the simulated and experimental stress–strain data. As a result, the deformation and the stress distribution of the structures under bending load, as presented in Figure 10C,D, could precisely identify the high-stress regions of the structures. The mechanical test results indicate that utilizing computationally generated bending test data in conjunction with actual measurements can provide a reliable method for assessing the mechanical deformation behavior of CP/MWCNT nanocomposite specimens. These innovative composites hold great potential for application in construction materials, contributing to green practices and reducing environmental impact. This is achieved by harnessing their thermoelectric properties to convert thermal energy losses into electrical energy [45]. As such, it is of outmost importance to ensure these materials’ structural integrity.

## 4. Conclusions

In the present study, the mechanical properties of cement paste reinforced with MWCNTs were examined both experimentally and numerically. A multiscale computational simulation approach supported the experimental results. For this scope, 3D RVEs were constructed using a random sequential adsorption algorithm, and finite element analyses were performed. Since the homogenized properties of the composite material were extracted, macro-scale analyses were also performed. The homogenized elastic and inelastic properties were in good agreement with the experimental results. Both in tension and compression, the addition of MWCNTs increased the strength of the cement paste, and increased deformation capacity under compression was noted. The computational analyses showed improved mechanical properties with an increasing concentration of MWCNTs, assuming ideal dispersion; however, practical limitations in dispersion lead to deteriorated mechanical properties, as observed in the experimental results in the case of higher MWCNT concentrations, but this was not captured by numerical modeling. Despite this, the multiscale computational approach accurately assesses the effective elastic properties and nonlinear constitutive behavior of CP/MWCNTs in both tension and compression, which could be valuable for optimizing cement-based composites for various engineering applications. Specifically, regarding the case of 0.1 wt% MWCNTs/CP, the numerical model exhibited a 13% underestimation of the Young’s modulus, a 1% overestimation of the compressive strength, and a 6% underestimation of the tensile strength as compared to the values measured through the experimental procedure. Further improvements could involve developing models to account for non-ideal dispersion and defects in the numerical simulations, leading to a more comprehensive understanding of the materials’ behavior.

## Figures and Tables

**Table 1:** Chemical composition of CEM I 42.4 R (Titan Cement Company S.A.) according to EN 197-1.

Chemical Composition and Properties	% by Mass	% *w/w*
Clinker	95–100	
Minor additional constituents	0–5	
Gypsum	-	
Sulfate content SO_3_		≤4.0
Chloride content		≤1.0
C_3_A in clinker		-

## Data Availability

The data presented in this study are available on request from the corresponding author.
